# Recent advances in engineering hydrogels for niche biomimicking and hematopoietic stem cell culturing

**DOI:** 10.3389/fbioe.2022.1049965

**Published:** 2022-11-24

**Authors:** Xiaochan Huang, Yuting Wang, Tianci Wang, Feiqiu Wen, Sixi Liu, Gerile Oudeng

**Affiliations:** ^1^ Department of Hematology and Oncology, Shenzhen Children’s Hospital, Shenzhen, Guangdong, China; ^2^ Shenzhen Children’s Hospital, China Medical University, Shenzhen, Guangdong, China

**Keywords:** hydrogels, hematopoietic stem cells, *in vitro* expansion, niche biomimicking, cell microenvironment

## Abstract

Hematopoietic stem cells (HSCs) provide a life-long supply of haemopoietic cells and are indispensable for clinical transplantation in the treatment of malignant hematological diseases. Clinical applications require vast quantities of HSCs with maintained stemness characteristics. Meeting this demand poses often insurmountable challenges for traditional culture methods. Creating a supportive artificial microenvironment for the culture of HSCs, which allows the expansion of the cells while maintaining their stemness, is becoming a new solution for the provision of these rare multipotent HSCs. Hydrogels with good biocompatibility, excellent hydrophilicity, tunable biochemical and biophysical properties have been applied in mimicking the hematopoietic niche for the efficient expansion of HSCs. This review focuses on recent progress in the use of hydrogels in this specialized application. Advanced biomimetic strategies use for the creation of an artificial haemopoietic niche are discussed, advances in combined use of hydrogel matrices and microfluidics, including the emerging organ-on-a-chip technology, are summarized. We also provide a brief description of novel stimulus-responsive hydrogels that are used to establish an intelligent dynamic cell microenvironment. Finally, current challenges and future perspectives of engineering hydrogels for HSC biomedicine are explored.

## 1 Introduction

Hematopoietic stem cells (HSCs) are self-renewing pluripotent stem cells. HSCs can differentiate into all types of blood cell in the body (Wilkinson et al., 2020). In the clinical treatment of hematopoietic diseases, transplantation of HSCs from healthy donors into patients can reconstitute hematopoiesis and the immune system in recipients (Chatterjee et al., 2021). There is a considerable demand for HSCs in clinical transplantation, gene therapy, and basic stem cell research (Wilkinson and Nakauchi, 2020). However, the lack of readily accessible HSC sources poses a barrier to the more widespread clinical and research use of these cells (Liu et al., 2022). The *in vitro* expansion of primitive pluripotent HSC populations promises to represent an effective solution to overcome this shortage (Liu et al., 2022).

HSCs used in transplantation are mainly derived from umbilical cord blood. These cells can be used with a relatively low risk of immune rejection, while the number of cells that can be recovered is limited (Ballen et al., 2013; Cohen et al., 2020).

In-depth studies regarding the molecular mechanism of self-renewal and differentiation in HSCs have explored various methods for HSCs expansion *ex vivo,* for example, supplementing hematopoietic stimulating factors, the addition of small molecule compounds into the suspension culture medium, and co-culture of HSCs and their supporting cells to maintain cell growth enhance the cellular connections (Zimran et al., 2021). However, it is difficult to are difficult to replicate cell–cell and cell–extracellular matrix (ECM) interactions that occur in the natural niche of the bone marrow (BM) using conventional two-dimensional (2D) culture methods (Langhans, 2018; Liu et al., 2022). To achieve a better *in vitro* hematopoietic microenvironment, research has focused on creating three-dimensional (3D) culture conditions *in vitro* (Huang et al., 2017). Hydrogel biomaterials have excellent biocompatibility, share some of the physical properties of the natural matrix, and provide a flexible solution to introduce biochemical and biophysical triggers. Ongoing efforts to establish an engineered BM-like niche for the *in vitro* expansion of HSCs is primarily focused on refining hydrogel-based culture methods (Yin and Cao, 2021).

**FIGURE 1:**
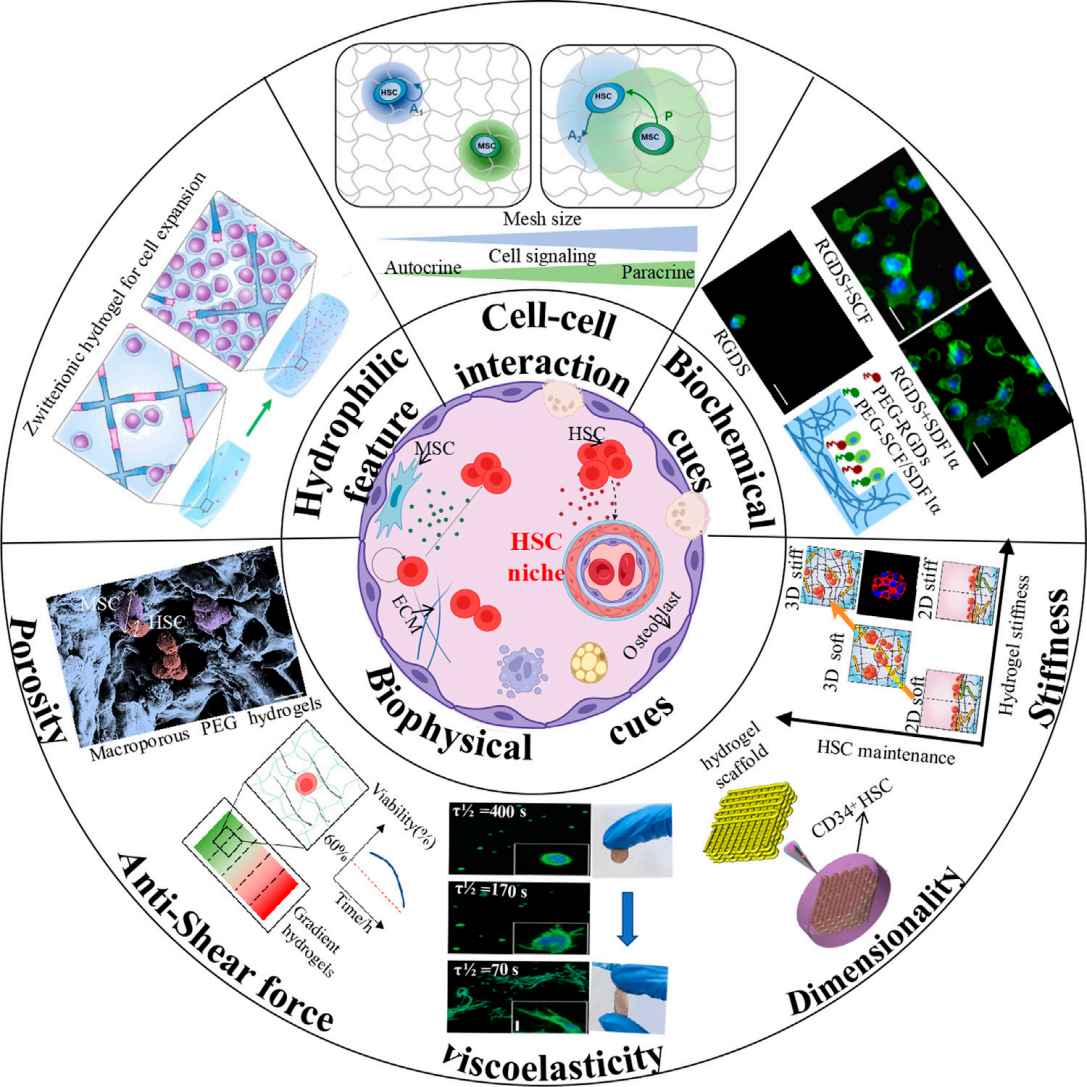
Schematic illustration of hydrogel engineering strategies for hematopoietic niche recapitulation. (Cell-cell interaction: (Gilchrist et al., 2019b) Copyright © 2019. Reproduced with permission from John Wiley and Sons; Biochemical cues: (Cuchiara et al., 2013) Copyright © 2019. Reproduced with permission from John Wiley and Sons; Stiffness: (Gvaramia et al., 2017) Copyright © 2017. Reproduced with permission from Elsevier; Dimensionality: (Zhou et al., 2020) Copyright © 2020. Reproduced with permission from Springer Nature; Viscoelasticity-Left: (Chaudhuri et al., 2016) Copyright © 2015. Reproduced with permission from Springer Nature; Viscoelaticity-Right: (Liang et al., 2022) Copyright © 2022. Reproduced with permission from American Chemical Society; Anti-shear force: (Mahadik et al., 2014) Copyright © 2013. Reproduced with permission from John Wiley and Sons; Porosity: (Raic et al., 2014) Copyright © 2014. Reproduced with permission from Elsevier; Hydrophilic feature: (Bai et al., 2019) Copyright © 2019. Reproduced with permission from Springer Nature.

This review summarizes hydrogel-based engineering methods used for recreation of the hematopoietic microenvironment for the purpose of the *in vitro* expansion of HSCs (Figure 1). We briefly describe the basic physiological characteristics of the hematopoietic niche, challenges culturing HSCs *ex vivo*, and recent culture approaches, followed by an overview of the engineering strategies used for replicating the natural hematopoietic niche. We also discuss new technologies for establishing an “intelligent microenvironment.” Finally, challenges and perspectives in the development of the hydrogel-based culture platforms for clinical application are evaluated.

## 2 The hematopoietic niche

In 1978, Schofield introduced the concept of the “niche”, which refers to the HSC microenvironment in the BM (Chatterjee et al., 2021). The niche provides indispensable factors for the self-renewal and differentiation of HSCs, controls the quiescence and stemness characteristics of HSCs, and regulates cell fate (Rasheed, 2022). In early studies, the hematopoietic niche was classified as the endosteal niche and perivascular niche based on the distribution of cell populations (Zhao and Li, 2016). The endosteal niche contains osteoblasts and osteoclasts at the endosteum. These cells regulate homing and the maintenance of quiescence in HSCs (Ingavle et al., 2019). The perivascular niche, formed in the vicinity of blood vessels, can promote the interaction of HSCs with endothelial, perivascular, and mesenchymal stem cells (MSCs) (Silberstein and Lin, 2013; Barnhouse et al., 2021). With increasing molecular mechanism studies, more and more cellular constituents have been identified in BM niche. Current research has showed that it is possible for different types of hematopoietic stem cells and progenitor cells (HSPCs) to have their own special niche created by multiple cell types. (Morrison and Scadden, 2014).

The HSC pool is highly heterogeneous in function and molecules it contains, which has increased the possibility that “specialized” niches are existed for “specialized” HSCs. This could also be inferred from the physiological distribution characteristics of HSCs. It has been reported that almost all HSC cells are in near range sinusoid vessels and are supported by the factors, such as Stem Cell Factor (SCF), C-X-C motif chemokine 12 (CXCL-12), and pleiotrophin, synthesized by leptin Receptor^+^ (LepR^+^) MSCs and endothelial cells (Christodoulou et al., 2020). Few HSCs are distributed in the BM, and <20% are located within 10 µ m from the BM intima (Lo Celso et al., 2009). These small HSC populations may be directly or indirectly regulated by factors near the bone surface (Cao et al., 2019; Zhao et al., 2019). In addition, some important factors for maintenance of HSCs are not synthesized in the BM, such as thrombopoietin (TPO), which is generated by the liver and transported to the BM through the blood (Nakamura-Ishizu et al., 2014). Therefore, the hematopoietic niche is composed of multiple components including different cells, biomolecules, and matrix, in which each component of HSCs has an important regulatory role.

### 2.1 Cellular compounds

#### 2.1.1 Osteoblasts

Osteoblasts are particularly common in the trabecular bone of the endosteum and are important in regulating HSC maintenance (Calvi et al., 2003; Zhang et al., 2003). Primitive HSPCs primarily home and engraft in the endosteal niche, which is rich in immature osteoblasts and osteoblastic progenitor cells (Levesque et al., 2010). Along with other cytokines, these immature osteoblasts secrete angiopoietin-1 (Arai et al., 2004), osteopontin (Stier et al., 2005), and CXCL12 to support HSC quiescence and self-renewal (Matteini et al., 2021). In contrast, mature osteoblasts induce the proliferation of HSCs (Choi et al., 2015).

Osteoblasts were one of the first non-hematopoietic BM cells reported to participate in HSPC regulation (Nilsson et al., 2001). Early studies showed that osteoblasts differentiated from human BM stromal cells could produce hematopoietic cytokines and support the HSC maintenance (Calvi et al., 2003; Zhang et al., 2003). More recent studies showed that conditional deletion of CXCL12 or SCF in osteoblasts did not affect the frequency of HSC in BM, which indicates that although these two factors are necessary niche factors for maintaining HSC, osteoblasts do not support the maintenance of HSCs through these niche factors (Greenbaum et al., 2013; Crane et al., 2017). It has been reported that osteoblasts can promote the maintenance of early lymphoid progenitor cells by secreting extremely low levels of CXCL12. It is worth mentioning that most lymphoid cells are located in the perisinusoidal niche in which they rely on CXCL12, and Transforming Growth Factor (TGF-β), synthesized by LEPR^+^ cells (Shen et al., 2021).

#### 2.1.2 Endothelial cells

Nutrient arteries entering the cortical bone divide into descending and ascending branches. These extend axially along the central cavity containing the BM, giving off radial branches. These enter the endosteal region of the cortical bone in a small network of Sca1^+^ arterioles and capillaries. These vessels anastomose with Sca1^-^ venous sinuses (approximately 50–100 µm in diameter). These venous sinuses drain into the central longitudinal vein running in the BM (Ramasamy, 2017; Owen-Woods and Kusumbe, 2022).

Sca1^+^ arterial endothelial cells are present in transitional vessels (or type H vessels), arterioles and arteries (Langen et al., 2017). Type H vessels represent a specific subset of capillaries localizing exclusively to the endosteal region of the BM. They enhance angiogenesis and osteogenesis by transducing signals to osteoprogenitor cells (Spencer et al., 2014). Both H-type vessels and arterioles exhibit low permeability, protecting HSCs from high levels of reactive oxygen species (ROS). This phenomenon prevents the overactivation of the progenitor cells that could cause the loss of stemness (Kunisaki et al., 2013; Silberstein and Lin, 2013; Spencer et al., 2014). Sca1^-^ sinusoidal endothelial cells represent the main endothelial components of L-type vessels (Langen et al., 2017). In contrast to the arteriolar system, around the sinusoids ROS levels are relatively high. This enhances the migratory capacity and activation of HSCs (Itkin et al., 2016). Nonetheless, some reports suggested that sinusoidal endothelial cells may also contribute to the maintenance of HSC quiescence (Acar et al., 2015).

#### 2.1.3 Mesenchymal stem cells

BM-derived MSCs have excellent potential for self-renewal and multilineage differentiation. They can form osteocytes, fat cells, cartilage, muscle cells, nerves, liver cells, and form the substrate cells supporting hematopoiesis (Pievani et al., 2021). MSCs secrete a variety of cytokines, hematopoietic and non-hematopoietic growth factors, and chemokines. Together, these modulate the BM microenvironment and regulate the proliferation and differentiation of HSCs. It appears that the secretion of CXCL12 by MSCs can induce the CXC12-CXCR4 mediated homing and retention of HSCs, while the production of stem cell growth factor (SCF) is responsible for cell survival and proliferation (Mannino et al., 2022). The simultaneous transplantation of MSCs and HSCs can promote HSCs homing, promote hematopoietic reconstruction, and reduce graft rejection (Jing et al., 2010). MSCs secrete multiple biomolecules by interacting with HSCs to support their growth and further to promote the hematopoietics. Meanwhile, MSCs inhibit the immunogenicity and immunomodulatory and reduce the incidence of graft-versus-host disease (GVHD). Co-transplantation of MSCs and HSCs has been applied in clinical and achieved positive results. Because MSCs are relatively easy to obtain without major ethical and legal issues their potential to support the *in vitro* expansion of HSCs can be easily studied.

#### 2.1.4 Adipocytes

Adipocytes are the most abundant cells in the BM of adults and are commonly seen in close contact with hematopoietic cells. Before birth the bone marrow cavity does not contain any fat cells but the amount of bone marrow adipose tissue (BMAT) increases with age and eventually occupies up to 50% of the BM cavity (Hardouin et al., 2014). Traditionally, adipocytes were thought to negatively regulate HSC function in the BM microenvironment (Naveiras et al., 2009). However, emerging evidence suggests that adipocytes may promote the maintenance of HSCs. Mattiucci (Matteini et al., 2021) demonstrated that BM-MSCs can differentiate into BM adipocytes expressing specific cytokines. Amongst these Interleukin-3 appear to support HSC survival.

#### 2.1.5 Progeny of hematopoietic stem cells

In addition to the stromal niche components, various progenies of HSCs including macrophages, neutrophils, regulatory T cells, and megakaryocytes, also participate in the regulation of the hematopoietic niche in the BM (Pinho and Frenette, 2019; Torres et al., 2022). Macrophages can regulate HSC migration; Bone marrow macrophages promote the retention of HSCs by regulating osteolineage cells and MSCs, thus antagonizing the excretion of HSC from BM mediated by SNS (Méndez-Ferrer et al., 2010; Chow et al., 2011). Macrophages clear senescent neutrophils and change the function of CXCL12 antibody reticular cells and retain HSCs in BM (Casanova-Acebes et al., 2013). Neutrophils derived from the bone marrow can promote endothelial cell regeneration after transplantation by secreting TNF, thus accelerating hematopoietic recovery (Bowers et al., 2018). Studies have shown that allogeneic hematopoietic stem cells co-locate with FoxP3^+^ regulatory T cells in the endothelial area after transplantation and can survive for 1 month without immunosuppressive drugs (Fujisaki et al., 2011; Hirata et al., 2018). However, depletion of Tregs cells leads to the rapid loss of allogeneic hematopoietic stem cells, indicating that FoxP3^+^ Tregs provide immune privilege for HSC (Fujisaki et al., 2011). Megakaryocytes maintain HSC quiescence by secreting CXCL4 (Bruns et al., 2014), TGF-β (Zhao et al., 2014), and TPO (Nakamura-Ishizu et al., 2014).

### 2.2 Cytokines

Cytokines are small protein molecules secreted by a variety of cells. By interacting with specific receptors they regulate the biological behavior of other cells. SCF, CXCL12, and thrombopoietin (TPO) are essential growth factors for HSCs (Yucel and Kocabas, 2018). SCF, also known as KIT ligand, exists in membrane binding and dissolving configurations (Asada et al., 2017). Both forms bind to the c-kit receptor, a tyrosine kinase, on the surface of HSCs, and promote HSC expansion. TPO is expressed mainly in the liver and kidneys and is a soluble factor necessary for the survival and proliferation of CD34^+^ HSPCs (Chatterjee et al., 2021). CXCL12 (also known as SDF-1) is secreted by several cells in HSC niche. CAR cells found in perivascular regions represent the main sources of CXCL12 (Mendelson and Frenette, 2014; Kosan and Godmann, 2016). The CXCL12/CXCR4 signaling cascade plays an essential role in the homing, mobilization, and retention of HSCs (Verma et al., 2020).

### 2.3 Neural regulation

There is evidence to show that the autonomic nervous system (ANS) and SNS can regulate the fate of HSCs (Maryanovich et al., 2018). ANS signals are important for the circadian mobilization of HSCs in the BM niche (García-García et al., 2019). SNS fibers respond to Granulocyte Colony-Stimulating Factors (G-CSF) and release norepinephrine, which activates β3-adrenergic receptors to control rhythmic changes in CXCL2 expression (Asada et al., 2013; Matteini et al., 2021). Non-myelinating Schwann cells promote HSC quiescence and maintenance by activating TGF-β and Suppressor of Mothers Against Decapentaplegic (SMAD)-induced signaling pathways (Mannino et al., 2022). However, studies on the regulating mechanism of sensory fibers on HSC behaviors in the BM niche are very limited in recent. One study reported that nociceptive receptors of the sensory neuropeptides lcitonin gene-related peptide could regulate G-CSF-induced HSC mobilization (Gao et al., 2021).

### 2.4 Niche extracellular environment

The ECM is a scaffold network that contains embedded HSCs and stromal cells. It is rich in collagen, laminin, and elastin, and contains a mixture of cytokines regulating proliferation and differentiation behaviors (Bello et al., 2018). This network is composed of two main classes of macromolecules: gel matrix components and fibrous protein components. The gel matrix components form gel-like structures composed of polysaccharides, amino sugars, and a range of protein constituents. In contrast, fibrous proteins primarily include collagen and elastic proteins and form a 2D or 3D structure with tunable stiffness and elasticity. This provides spatial support for the survival of HSCs and regulates their growth through interactions with integrin molecules present on the surface of HSCs (Chatterjee et al., 2021). The most studied fibrous proteins of the natural niche are fibronectin and laminin, which can bind to integrins and regulate the migration and homing behavior of HSCs. For example, studies have shown that BM laminins promote adhesion and migration of human HSCs through interaction with the integrin α_6_ receptor for homing (Gu et al., 2003). Stiffer endosteal environments support the differentiation of HSCs into primitive myeloid progenitors, while softer substrates promote differentiation along the erythroid lineage. The adherence and motility of HSPCs is significantly higher on harder surfaces.

## 3 Hematopoietic stem cell culture *ex vivo*

### 3.1 Challenges of hematopoietic stem cell culture *ex vivo*

Two main types of HSCs were defined: long-term (LT)-HSCs, which are deeply quiescent in niches to maintain self-renewal and multilineage differentiation potential throughout their lives, and short-term (ST)-HSCs, which are maintained in more active niches with limited self-renewal potential (Choi et al., 2015). The main goal of *ex vivo* culture of HSCs is to expand the number of HSCs and HSPCs with a high capability of self-replicating and self-maintenance. However, few normal HSCs are in the cell cycle and undergo asymmetric mitosis. The asymmetric mitosis of HSCs is highly self-replicating and self-sustaining. After several mitoses, the cells stop entering the S phase and turn to the quiescent phase G_0_, therefore, it is difficult to effectively expand them *ex vivo*. If HSCs are induced to undergo symmetrical mitosis through gene mutation, that is, the cells expand indefinitely without differentiation, this will inevitably lead to malignant transformation of the cells, with a loss in the ability to self-renewal and self-maintenance. During cell culture, supplementation with inappropriate cytokines or long-term culturing can promote both proliferation and differentiation of cells and destroy the asymmetric mitosis of the cells. Therefore, long-term culture may obtain a large absolute number of CD34^+^ cells or nuclei cells; it also easily to lead to the differentiation and exhaustion of the early progenitor cells and only produces numerous HSPCs without hematopoietic reconstitution function.

Heterogeneity of HSCs is another important reason for the difficulty in their expansion *in vitro*. *Ex vivo* expansion methods of HSCs reported in the literatures are often quite different. Even under the same conditions of cytokine combination, the expansion ability and transplantation ability of cells are still significantly differ. The heterogeneity of HSCs also makes it challenging to understand the comprehensive signaling mechanism that defines the balance between homeostasis and self-renewal of among cells. Recent evidence has added new insights into the plasticity of BM niche and mitochondrial network, which synergistically determine the fate of HSCs. However, these new discoveries also increase the complexity and introduce a highly dynamic interrelationship in studies of HSC fate. Therefore, research on the complex mechanisms controlling the fate of HSCs remains limited.

### 3.2 Recent approaches to hematopoietic stem cell culture *ex vivo*

Compared with haplotype hematopoietic stem cell transplantation (haplo HSCT), umbilical cord blood stem cell transplantation (UCBT) can be easily obtained from CB resources. UCBT has high tolerance to human leukocyte antigen HLA mismatch, a high colony forming potential of HSCs and HSPCs, and a low incidence and recurrence rate of graft versus host disease (GVHD) following transplantation. Furthermore, HSCs are ideal vector cells for gene therapy. The long-term self-renewal and self-maintenance abilities of HSCs are conducive to the long-term expression of the target therapeutic genes *in vivo*. However, insufficient amounts limit the clinical application. *Ex vivo* expansion of HSCs is the basis both for the clinical promotion of UCBT and gene therapy. Therefore, many efforts have been focused on improving HSCs expansion methods.

Supplementing with the hematopoietic stimulators is a typical method for HSC expansion. Cytokines are one of the first molecular stimulators for HSC expansion *ex vivo*. After 5–8 days of culturing of human CB CD34^+^CD38^−^ cells in serum free medium containing Fms Related Tyrosine Kinase 3 (Flt-3), SCF, Interleukin (IL)-3, IL-6 and G-CSF, the number of colony forming units (CFU) increased 100 -fold, and the number of long-term culture-initiating cells (LTC-IC) increased 4-fold (Conneally et al., 1997). The addition of Fibroblast Growth Factor 1 (FGF1) and FGF2 to the serum free medium during the co-culture of HSCs and osteoblast supported the expansion and regeneration of HSCs *in vitro* (Yeoh et al., 2006). Notch signaling pathway is an important pathway for HSC fate and lymphocyte generation. An engineered Delta-like ligand Delta1^Ext−IgG^ (DXI) was used to activate the Notch signal pathway in human CB CD34^+^ cells to achieve clinically significant *in vitro* expansion of ST-HSPCs with and shorten the implantation time of neutrophils following transplantation (Delaney et al., 2005; Delaney et al., 2010). In addition, TNF Superfamily Member 15 (TNFSF15), the Wnt signaling pathway and Prostaglandin E2 (PGE2) were used *ex vivo* to expand the long-term reconstruction ability of HSPCs (Ding et al., 2020).

The addition of small molecule compounds such as copper chelator tetraethylenediamine (TEPA), nicotinamide, stem regin-1 and valproic acid, which simultaneously expand ST- and LT-HSCs, provide effective schemes for HSC amplification *in vitro* (Zimran et al., 2021). U1M171, an HSC agonist, is one of the most promising small molecules for HSC expansion in recent years (Fares et al., 2014). In 2014, Guy Sauvageau and Peter W zandstra (Fares et al., 2014; Pabst et al., 2014) were the first to show that UM171 can significantly expand umbilical CB stem cells and enhance multilineage hematopoietic reconstruction in rats. In 2019, the team published Phase I/II clinical trial results (NCT02668315) on the safety and feasibility of a single UM171 expanded umbilical CB graft in The Lancet Haematology (Cohen et al., 2020). The minimum dose of rapidly implanted umbilical CB cells in this clinical trail was 0.52 × 10^5^ CD34^+^ cells, which has relatively low chronic GVHD and recurrence risk due to rapid T cell reconstruction.

MSCs are the main cell component of hematopoietic microenvironment and plays an important role in the growth, proliferation, and differentiation of HSCs. MSCs support and promote hematopoiesis by interacting with HSCs and secreting various cytokines, or expressing, and through the expression of adhesion molecules and ECM proteins related to adhesion and homing of HSCs. *In vitro* experiments have shown that MSCs plays an important role in the maintenance and expansion of CD34^+^ BM cells, but do not affect the erythroid differentiation ability (Lau et al., 2017). At present, great progress has been made in the research of MSC-promoting hematopoietic reconstitution following HSCT. MSC and HSC co transplantation supports the growth of HSCs and significantly improves megakaryocyte and platelet formation. In addition, MSCs may also play a role in epigenetic regulation during HSC differentiation (Zakrzewski et al., 2019).

Static culture systems, such as culture plates and flasks, generally require repeated processing of HSCs regarding fluid change. Furthermore, due to ineffective mixing, the non-homogeneous of compounds may result in differences in the initial stimulation of cultured cells. The continuous perfusion bioreactor is a fully closed dynamic culture automation device to create 3D constructed microenvironments with different cellular or biomechanical features (Bhaskar et al., 2018). For example, Martin’s group developed a perfusion bioreactor system analogue, which partially recapitulated the structural and compositional features of the human osteoblastic niche. Human purified CB-derived were cultured in the bioreactor; after 1 week culture, number of the CD34^+^ cells and colonies numbers significantly increased. Perfusion bioreactor systems provided important evidence for dynamic control of the expansion quality during *ex vivo* HSC culture (Bourgine et al., 2018).

The hematopoietic niche is composed of biophysical and biochemical factors, which play a key role in the static, self-renewal, implantation, migration, differentiation, and other behaviors of HSCs. HSCs in the niche are affected by the mechanical force matrix, physical or chemical stimulations from stromal cells and the environment, as well as the cytokines distributed in the 3D niche space. Biomaterials with adjustable physical, mechanical, and chemical properties can easily be modified by different biological molecules, which opens up new opportunities for controlling the bionics of the niche. Compared with inorganic or metal biomaterials, hydrogels are organic materials with excellent biocompatibility. *In vivo*, the hematopoietic niche is a 3D environment dominated by hydrophilic and amphiphilic cell membrane lipids. Hydrogels have good hydrophilicity and rich functional groups; it is easy to modify the hydrophobic groups and various biomolecules. Their controllable physical and chemical characteristics, such as hardness, porosity, elasticity, and functionalization of key cytokines, can be beneficial in creating an environment closer to the niche.

## 4 Engineering hydrogels based on niche physiology

When HSCs detach from their niche microenvironment, the cells easily exhaust and differentiate. 2D culturing is not consistent with the spatial feature of natural niches. The intricated chemical and physical cues inside niche are crucial to regulate HSCs behavior. Therefore, 3D culturing of HSCs has been extensively researched recently; hydrogel biomaterials with excellent biocompatibility have been gradually employed for establishing a 3D environment for HSCs. (Yan et al., 2022) (Maji and Lee, 2022).

Hydrogels with good hydrophilicity capacity can swell in aqueous media, minimize non-specific protein adsorption, and inhibit collagen capsule formation, thus preventing undesirable differentiations of HSCs (Zhang et al., 2015; Erathodiyil et al., 2020). In addition, the hematopoietic niche consists of a series of ECMs and interacting components (Winkler et al., 2020). Engineered hydrogels can be developed to contain interconnected polymer networks to spatially mimic the ECM networks and the cell–cell contacts in the natural niche. Stiffness can be achieved by regulating the degree of polymer cross-links (Rosales and Anseth, 2016; Tang et al., 2021; Maji and Lee, 2022). Such structures render hydrogels high permeable, supporting nutrient exchange between cells and the environment (Liu et al., 2019).

To better replicate cell–matrix interactions and improve the responsiveness of hydrogels to microenvironmental changes, they can be chemically and mechanically modified. These modifications enable the production of hydrogels with adjustable viscoelastic, mechanical, degradation, and cell adhesion characteristics, as desired (Zhang and Khademhosseini, 2017; Prince and Kumacheva, 2019; Caldwell et al., 2020; Liang et al., 2022). Aside from the provision of an artificial niche in cell cultures, hydrogels can also be used in the experimental studies of cellular behaviors and cell–material interactions (Costa et al., 2022). Various components can be introduced into engineered hydrogels to help the mimic the hematopoietic niche for the expansion of HSCs and the regulation of cellular behavior. These modifications of hydrogels are examined in the following sections. Recent engineering hydrogel strategies of the biomimetic hematopoietic niche and *in vitro* cultures are summarized in Table 1. Information on the characterization and functional assessments of cultured HSCs on these hydrogel-based studies are supplemented in Table 2.

**TABLE 1:** Engineering hydrogel strategies of biomimetic hematopoietic niche and *in vitro* HSC culture.

Hydrogel composition	Engineering strategies	Key findings	Comments	Ref.
Puramatrix gel	3D coculture of human BM-derived CD34^+^ with human placenta-derived MSCs	Hydrogel-based 3D culture of MSCs mimicked a functional hematopoietic niche for quiescent HSC maintenance and simultaneous multilineage hematopoiesis *via* the CXCR4-SDF1α axis, integrin beta1-mediated adhesion interactions, and a hypoxia gradient.	This study illustrates the specific pathways that supports the quiescence and pluripotent capability of HSCs in hydrogel-based MSC coculture system.	Sharma et al. (2012)
Collagen	3D coculture of HSCs with MSCs derived either from BM or UC	MSCs supported the migration of HSCs into the collagen hydrogel and promoted clonal expansion of HSC populations with higher ration of primitive CD34^+^CD38^−^ phenotype	3D coculture system of HSCs and BM-MSCs in collagen scaffold resembles the endosteal niche and allows dissecting two subpopulations of HSCs.	Leisten et al. (2012)
PEG-DA	Photopolymerization; RGDS, SCF, and SDF-1α were covalently immobilized onto the surfaces of hydrogels	Cell adhesion and spreading was enhanced by immobilizing biomolecules on hydrogel surfaces.	This study does not demonstrate that the hydrogel-based culture system can promote the *in vitro* expansion of HSCs and maintaining pluripotent potential.	Cuchiara et al. (2013)
Collagen	Microfluidic; Opposite-gradient populations of HSCs and osteoblasts	The hydrogel construct with stable multicellular gradients was created in a manner independent of cell size and hydrogel density and enabled the modulation of microenvironmental signals on HSC fate.	The microfluidic generation of gradient hydrogels with heterotypic microenvironment helps to study the impact of microenvironmental signals on HSC fate.	Mahadik et al. (2014)
Type I collagen gel	Microfluidic BM on-a-chip; photolithography; Engineered BM was formed in a PDMS device *in vivo* and then cultured in a microfluidic system	The BM on-a-chip replicated a functional hematopoietic niche *in vitro*, supporting HSCs in normal *in vivo*-like proportions for at least 1 week.	The *in vivo* engineering strategy enables the reconstitution of hematopoietic niche physiology and function restoration of natural BM.	Torisawa et al. (2014)
Methacrylamide-functionalized gelatin (GelMA) hydrogel	Microfluidic; PEGylated SCF were covalently immobilized within GelMA hydrogel *via* photochemistry	The GelMA hydrogels with immobilized SCF selectively maintained primitive HSCs and generated more GEMM colonies while soluble SCF induced lineage specification.	The study shows the effect of the local presentation of soluble vs. matric-immobilized biomolecules on HSC proliferation and lineage differentiation.	Mahadik et al. (2015)
PEG-DA	Photopolymerization; SCF, IFNγ, RGDS and connecting segment I were conjugated into hydrogels.	HSCs expanded 97-fold and 104-fold with the supplement of SCF and IFNγ respectively, and retained good differential capability.	Bioactive molecules modified on hydrogels promotes the recapitulation of functional HSC niche.	Cuchiara et al. (2016)
Collagen	Primary HSCs were cocultured with lineage positive (Lin^+^) niche cells on collagen hydrogels with varying hydrogel densities and HSC: Lin^+^ ratios	HSC-generated autocrine signals that dominate in the low-diffusive hydrogel matrix promoted the proliferation of early hematopoietic progenitors, while niche cell-generated paracrine signals that dominate in the high-diffusion hydrogel environment enhanced myeloid differentiation.	The effect of collagen hydrogels on autocrine and paracrine signaling between HSCs and niche cells depends on the hydrogel diffusivity and niche cell density.	Mahadik et al. (2017)
Methacrylated hyaluronic acid (HAMA)	*In-situ* radical crosslinking; HA/GO hydrogels were fabricated by methacrylated hyaluronic and methacrylated graphene oxide (GO).	HA/GO hydrogels exhibited excellent water absorption ability and elastic moduli. HA/GO-0.05 hydrogel showed a higher expansion of CD34^+^ cells compared to HA, HA/GO-0.1, HA/GO-0.2.	The incorporation of nanomaterials can enhance the biophysical properties of hydrogels to promote the proliferation of HSCs.	Guo et al. (2017)
HAMA-methacrylated gelatin	Photo-crosslinking; SCF was physically encapsulated within HA/gelatin double network (HGDN) hydrogel to achieve a slow-release rate	The HGDN hydrogels achieved a sustained release of SCF to reduce the consumption of SCF. This cost-effective protocol could support the proliferation of HSCs and generate more multipotent colony-forming units.	SCF-loaded HGDN hydrogel provides a cost-effective *ex vivo* culture strategy for HSCs	Zhang et al. (2018)
Star-shaped poly (carboxybetaine acrylamide)	Click reaction; modified with polypeptide crosslinkers containing alternating K and E amino acid sequences and metalloproteinase	The zwitterionic hydrogel showed excellent hydrophilicity and ROS scavenging capability, leading to a 73-fold increase in long-term HSCs and hematopoietic reconstitution in mice up to 24 weeks.	The zwitterionic hydrogels with excellent hydrophilicity and anti-fouling properties hold great promise in realizing the long-term *in vitro* expansion of multipotent HSCs.	Bai et al. (2019)
Hyaluronic acid/carbon nanotubes antioxidant hydrogel	Photo-crosslinking; functionalized CNTS were incorporated into hydrogels	The HA/CNT hydrogels exhibited concentration-dependent antioxident activities to significantly enhanced the expansion of HSCs.	The antioxidant hybrid hydrogels provide a microenvironment with low ROS level for *in vitro* proliferation of HSCs.	Zhang et al. (2019b)
Thiolated HA, thiolated gelatin, PEGDA crosslinker	Microfluidic BM on-a-chip; 4 niche cell populations were encapsulated in the ECM-derived hydrogel, each in its own chamber of a microfluidic device	The microfluidic BM on-a-chip was deconstructed into four distinct but integrated niche constructs. Healthy and malignant human HSCs showed selective homing to specific niche on this device.	It provides a tool to study the interactions of normal and malignant HSCs with niche cells, which facilitate to delineate the HSC-niche signaling pathways.	Aleman et al. (2019)
Gelatin-alginate	3D bioprinting; hydrogel scaffold for HSCs and UC-MSCs coculture	CD34^+^CD38^−^ cells expanded 33.57-fold and CD34^+^CD184^+^ cells expanded 16.66-fold after 10 days of coculture with engineered scaffolds	The study demonstrates the feasibility of 3D bioprinting in constructing HSPC and MSC coculture environments.	Zhou et al. (2020)
Collagen	Microfluidic BM on-a-chip; Top channel: human CD34^+^ cells and BMSCs in a fibrin gel; bottom channel: HUVECs	The vascularized human BM chip supported the differentiation and maturation of multiple blood cell lineages while improving the maintenance of CD34^+^ progenitors.	BM on-a-chip with physiological function can be utilized to study the pathophysiology of hematological diseases and BM toxicity of drugs.	Chou et al. (2020)
PEG-DA/MA	Cryogelation; Iron nanoparticle are embedded in polymer solutions by pulsed laser ablation in liquids	PEG-iron nanocomposite hydrogels showed constant iron release to enhance erythroid differentiation.	This study proposes a strategy to guide erythroid differentiation in an *ex vivo* HSC culture model	Brandle et al. (2020)
Alginate-Puramatrix	Chemical crosslink; droplet-based microfluidics; Double layered hydrogel beads (inner: MSCs in alginate; outer: HSCs in Puramatrix) were cultured in DMEM with 10% FBS without additional supplements.	Human BM-derived CD34^+^ cells survived, maintained, and expanded over 8 weeks in microdroplet.	This study presents a novel and economical protocol for the encapsulation, maintenance, and expansion of primary HSCs.	Carreras et al. (2021)

**TABLE 2:** Some hydrogel-based studies for *in vitro* HSC culture and expansion.

HSC source	HSC phenotype	Culture maintenance	Functional assessment/other HSC-related analysis	Ref.
Human BM HSCs	CD34^+^	7 days	*In vitro* CFU assay, Long-term culture initiating cell assay, *In vivo* study of secondary transplantation in NOD/SCID mice	Sharma et al. (2012)
Human UCB-HSCs	CD34^+^	14 days	Viability assay, cell division tracking, flow cytometry, histomorphological analysis	Leisten et al. (2012)
Murine 32D cells, myeloid progenitor cells	—	6 days	Evaluation of cell adhesion and spreading	Cuchiara et al. (2013)
Murine BM HSCs	LSK HSCs	3 days	Viability assay	Mahadik et al. (2014)
Murine BM HSCs	LSK HSCs, progenitor cells (Lin^−^Sca1^+^, Lin^-^cKit^+^, Lin^−^CD34^+^, Lin^−^CD135^+^)	7 days	Viability assay, BM transplant in irradiated mice	Torisawa et al. (2014)
Murine BM HSCs	LSK HSCs	7 days	Viability assay, *in vitro* CFU assay	Mahadik et al. (2015)
Murine BM HSCs	LSK HSCs	14 days	Viability assay, CFU assay	Cuchiara et al. (2016)
Murine BM HSCs	LSK HSCS	2 days	Viability assay, CFU assay	Mahadik et al. (2017)
Human UCB HSCs	CD34^+^	10 days	CFU assay	Guo et al. (2017)
Human UCB HSCs	CD34^+^	14 days	CFU assay	Zhang et al. (2018)
Human UCB- and BM- derived HSCs	CD34^+^CD38^−^CD45RA^−^CD49f^+^CD90^+^	24 days	*In vitro* CFU assay, *in vivo* study of secondary transplantation in NSG mice, Limiting dilution analysis	Bai et al. (2019)
Human UCB HSCs	CD34^+^	10 days	Viability assays, CFU assay	Zhang et al. (2019b)
Human BM HSCs	CD34^+^	5 days	Viability assay	Aleman et al. (2019)
Human UCB HSCs	CD34^+^	10 days	Viability assay	Zhou et al. (2020)
Human peripheral blood-derived HSCs	CD34^+^	14 days	CFU assay	Chou et al. (2020)
Human UCB HSCs	CD34^+^	7 days	Flow cytometric analysis	Brandle et al. (2020)
Human BM HSCs	CD34^+^	8 weeks	Viability assay	Carreras et al. (2021)

### 4.1 Polymers for matrix networks

**FIGURE 2:**
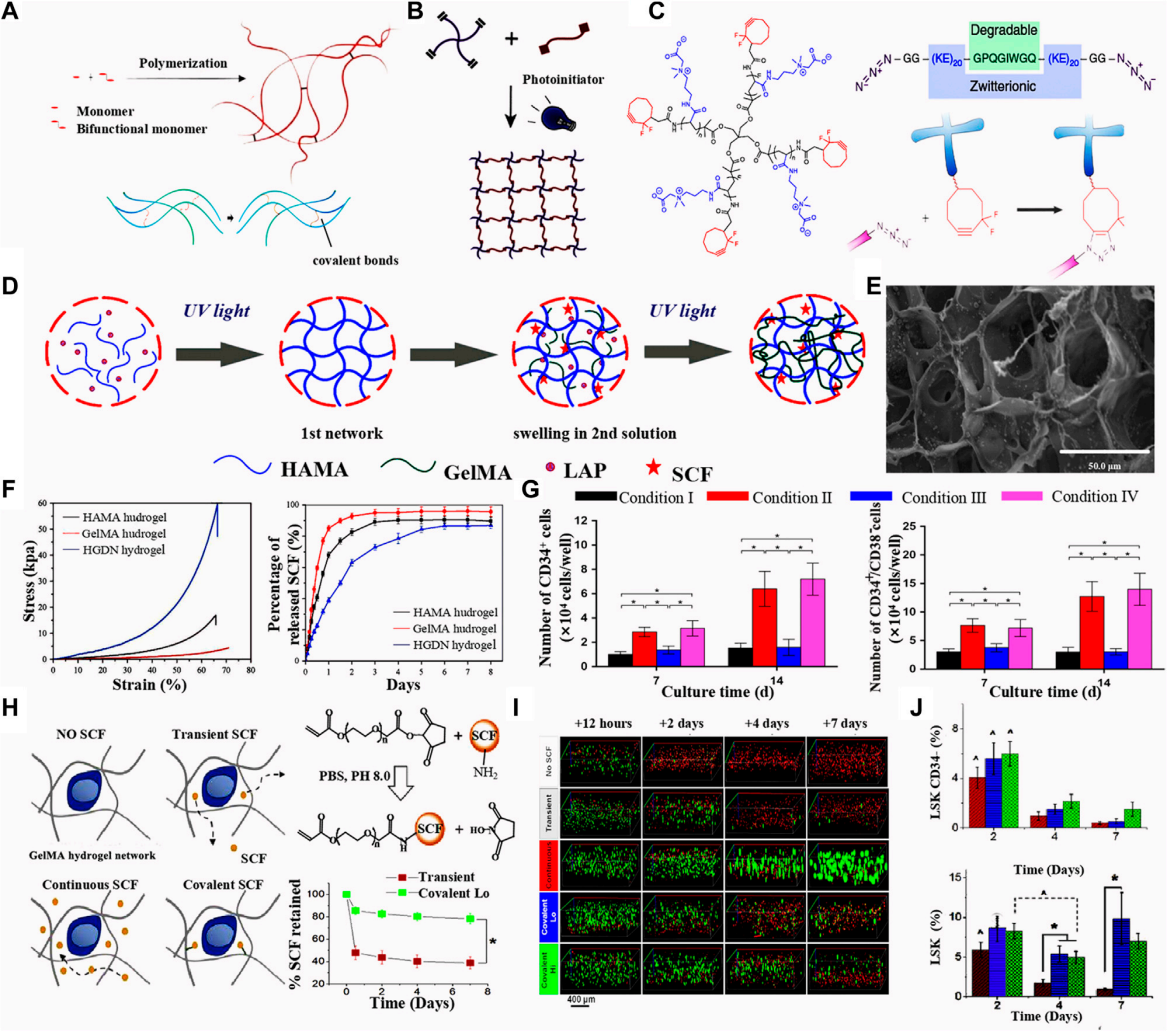
**(A)** Scheme of chemical crosslinking of hydrogel. Upper: (Yin and Cao, 2021) Copyright © 2021. Reproduced with permission from Elsevier. Lower: (Zhang and Khademhosseini, 2017) Copyright © 2017. Reproduced with permission from The American Association for the Advancement of Science. **(B)** Scheme of photo crosslinking. (Nezhad-Mokhtari et al., 2019 ) Copyright © 2019. Reproduced with permission from Elsevier. **(C)** Schemes of poly (carboxybetaine acrylamide) with a star-shape; polypeptide crosslinkers with KE sequences as the metalloproteinase degradable sites; The hydrogel synthesis basing on the step-growth polymerization mechanism of Huisgen cycloaddition. (Bai et al., 2019) Copyright © 2019. Reproduced with permission from Springer Nature. **(D)** Fabrication of the HGDN double network hydrogel and the physical loading of SCF. **(E)** Scanning electron microscopy images of the SCF-loaded HGDN double network hydrogel. **(F)** Stress-strain curves and SCF releases analysis of the HAMA, GelMA and HGDN hydrogels. **(G)** Percentages of CD34+ and CD34+CD38- phenotype of cells culture for 7 and 14 days. (Zhang et al., 2018) Figure 2D-G: Copyright © 2017. Reproduced with permission from John Wiley and Sons. **(H)** Different methods or SCF modification in hydrogels (left); SCF release curve of the transient and covalent modification of SCF. **(I)** Florescence images of the phenotype of cells cultured hydrogels with different release mode of SCF. **(J)** Percentage of LSK and CD34+ phenotype of cells culture in the SCF-loaded hydrogels with different culture time. (Mahadik et al., 2015) Figure 2H-J: Copyright © 2015. Reproduced with permission from Elsevier.

Hydrogels are fabricated by covalently or non-covalently cross-linking polymer chains dispersed in an aqueous solution. These 3D hydrogels are constructed by chemical cross-linking (Zhang and Khademhosseini, 2017; Yin and Cao, 2021) (Figure 2A), photo cross-linking (Nezhad-Mokhtari et al., 2019) (Figure 2B) and atom-transfer radical polymerization (Bai et al. (2019)) (Figure 2C). Depending on the source of polymers, hydrogels are classified into natural, synthetic, and hybrid polymers (Maji and Lee, 2022). As the name indicates, natural hydrogels contain polymers derived from biological sources. These matrices commonly contain alginate, hyaluronic acid (HA), chitosan, collagen, gelatin, and fibrin. Natural polymers are inherently biocompatible with living cells (Bao et al., 2019; Jiang et al., 2020; Peers et al., 2020); however, their practical use is limited by batch-to-batch compositional inconsistency, poorly controlled polymerization kinetics, and chain configuration (Echalier et al., 2017). These limitations can be overcome by using of bioengineered versions of natural polymers. These synthetic alternatives facilitate the controlled polymerization of the hydrogels (Zhao et al., 2016; Zhan et al., 2022). Polyethylene glycol (PEG), polylactic acid, polyvinyl alcohol, and polyurethane are synthetic polymers commonly utilized in tissue engineering and *in vitro* cell culture (Gjorevski et al., 2016). Hybrid hydrogels combine the best features of natural and synthetic polymers. Most research and practical use applications rely on these hybrid systems (Afewerki et al., 2019). Changing the composition and ratio of the incorporated polymers affects both the biocompatibility and mechanical properties (e.g., stiffness and degradation time), thus influencing cell fate and behavior (Wolf et al., 2015; Zhang et al., 2016). For example, keratin/PEG composite hydrogels exhibit a high compressive modulus of 45 kPa and long-term stability in physiological buffer solutions and cell culture media (Yue et al., 2018).

### 4.2 Regulation of bioactive molecules

The BM microenvironment contains various biologically active components, such as cell adhesion molecules, growth factors, cytokines, enzymes, and other signaling molecules. These represent specific signaling pathways and regulate diverse cellular processes, including proliferation, differentiation, migration, and apoptosis (Hastings et al., 2019; Winkler et al., 2020). Non-modified synthetic polymers and most natural polymers (e.g., alginate and chitosan) lack these bioactive molecules (Maji and Lee, 2022). Therefore, considerable effort has been devoted to developing hydrogel polymers containing biomolecules necessary for the creation of a cellular microenvironment that enables the precise control of cellular behaviors (Caldwell et al., 2020). These biomolecules can be incorporated by non-covalent means, such as physical entanglement (Bovone et al., 2021), electrostatic interactions (Sivakumaran et al., 2013; Wang et al., 2018), or through covalent tethering (Zhang and Khademhosseini, 2017; Bovone et al., 2021). Incorporating biomolecules *via* non-covalent weak interactions is more conducive towards the controlled release of nutrients and regulatory molecules.

Zhang group (Zhang et al., 2018) fabricated a HA/gelatin double network (HGDN) hydrogel that is physically loaded with stem SCF. The HA methacrylate (HAMA) hydrogel was fabricated by pouring the pre-gel HAMA solution and the photo-initiator phenyl-2,4,6-trimethylbenzoyl phosphonate lithium (LAP) into a round mold, which was then irradiated solution with ultraviolet light for a fast cross-linking. The HAMA network was then immersed in the GelMA solution with LAP until swelling equilibrium was reached and then photo-cross-linked to form a double network Figure 2D. This double network hydrogel is combined a fragile network and a double network and exhibits high mechanical strength and sustained release of SCF (Figure 2E, F). After 14 days of culturing, the total number of nucleated cells in the SCF-loaded HGDN hydrogel was approximately 3-fold higher than that of cells in the non-loading hydrogel, and the proportion of CD34^+^ CD38^−^ cells was slightly higher in the non-SCF treated group (Figure 2G). A CFU assay showed SCF-loaded HGDN hydrogels had more primitive CFU-GEMM (granulocyte, erythrocyte, monocyte, megakaryocyte) phenotype compared to the cells in plated with SCF. The HGDN hydrogel retained the SCF release for 6 days. Chemical incorporation of biomolecules through covalent, ionic, or metallic binding is widely used in the construction of 3D hydrogel networks (Xin et al., 2019; Chimene et al., 2020). Mahadik et al. (2015) demonstrated a methacrylamide-functionalized gelatin (GelMA) hydrogel covalently modified with SCF for culturing of primary HSCs isolated from the BM of mice. SCF was linked to the PEG- N-hydroxysuccinimide (NHS) ester and reacted with GelMA hydrogel precursor in solution (Figure 2H). When comparing the performance of the continuous SCF and covalent SCF in GelMA hydrogels, continuous SCF greatly increased the cell number after 7 days of culture and quickly reduced the cell number in longer culture, while reducing the maintenance of the initial LSK phenotype and primitive CD34^−^ LSK cells (Figures 2I, J). In contrast, covalent SCF showed increased maintenance of the 2 cell populations. At day 7, CFU colonies had increased for cells treated with covalent SCF, and the cell expansion reached 3 which was higher than that of day 1.

Cuchiara et al. (2013) covalently immobilized the cell adhesion peptide RGDS, the cell growth factor SCF, and the stromal derived factor 1a (SDF1α) on the surface of a PEG hydrogel to control the adhesion and spreading of HSCs. An anchorage-independent hematopoietic cell line, named 32D clone 3 cells were seeded into the PEG-diacrylate (DA) hydrogel wells modified with the above compounds. The number of adherent 32D cells was higher in the 25 μg cm^−2^ CF and SDF1α modified hydrogel well compared with other concentration, in addition to a larger adhere area (450 μm^−2^). Sustained activation of CXCR4 receptor in the 32D cells shows the effect of biomolecules on 32D cell migration in the modified PEG-DA hydrogel. A concept of biological cues introduced into the hydrogel well for HSC culture was revealed, while experiment on the measurement of more HSC biomarkers has been limited.

**FIGURE 3:**
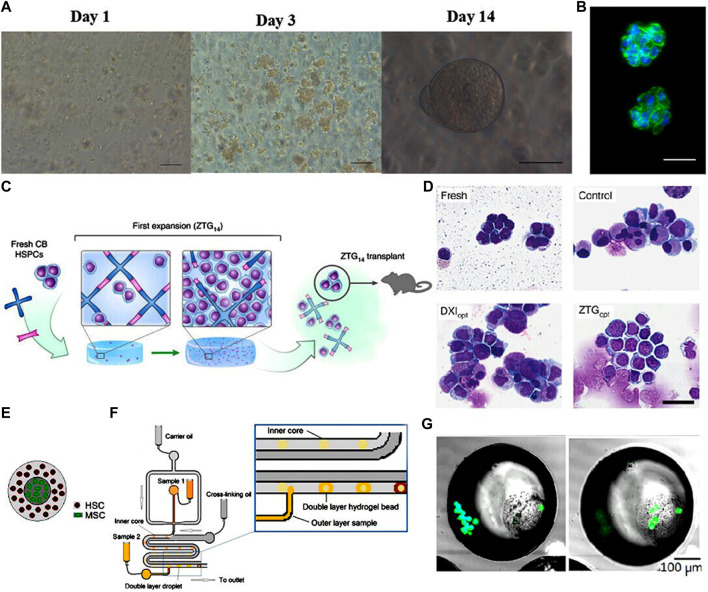
**(A)** Phase contrast images of HSCs encapsulated within bioactive PEG hydrogels at days 1, 3, and 14 showing the proliferation and round shape retention of HSCs. Scale bar: 100 mm (day 1, day 3), 50 mm (day 14). **(B)** DAPI (blue) and phalloidin (green) staining of HSCs after 14 days expansion showing the cell morphology retention and gel structure integrity. Scale bar: 25 mm. (Cuchiara et al., 2016) Figure 3A, B: Copyright © 2015. Reproduced with permission from John Wiley and Sons. **(C)** Schematic illustration of the zwitterionic hydrogels with superhydrophilicity and volumetric swelling. **(D)** Photomicrograph of Wright-Giemsa-stained HSCs showing the significant expansion of the primitive HSC population in zwitterionic hydrogels. Scale bar: 30 μm. (Bai et al., 2019) Figure 3C, D: Copyright © 2019. Reproduced with permission from Springer Nature. **(E)** Schematic representation of the double layered hydrogel bead with HSCs on the outer core and MSCs on the inner core. **(F)** Schematic illustration of the double layered structure fabrication process using droplet-based microfluidic. **(G)** Fluorescent staining of viable MSCs and HSCs show that both types of cells are successfully encapsulated in the double layered structure. (Carreras et al., 2018) Figure 3E-G: Copyright © 2018. Reproduced with permission from Elsevier.

This research group also fabricated a HSPC culture substrate with the derivatives of PEG-DA hydrogels. PEG-DA hydrogel wells were fabricated *via* a microfabrication patterning technology assisted by a photomask and photoresist. Proteins and peptides including the key cytokines, SCF, interferon-g (IFNg) and RGDS connecting segment 1, were chemically modified onto PEG with certain ratios to form the PEG-protein and PEG-RGDS composite. The composite was immobilized on the well surface *via* a photo-induced linking reaction. C-kit^+^ cells collected from mouse BM cells were cultured in the PEG-DA hydrogel wells. After 2-week culture, the cell number increased by 11.6–19.0% for cells 3D encapsulated in hydrogel, which is higher than that in 2D culture, and 53.8% cells remined c-kit^+^ without an observed loss. Encapsulated c-kit^+^ cells could form all colonies which had similar results to that of 2D culture. However, 3D culture exhibited a larger number of multilineage HSPC-derived colonies than that of 2D culture. These experiments demonstrated that cytokines encapsulated in a 3D scaffold better replicated the cell–matrix interactions and showed advantages in controlling diffusion in cultures designed for LT-HSC expansion compared with the 2D hydrogel (Cuchiara et al., 2016) (Figures 3A,B). SCF is a key component of the native HSC niche and needs to be continuously and abundantly supplied in the medium when HSCs are cultured *in vitro*. Mahadik et al. (2015) reported the use of a composite hydrogel created by chemically immobilizing SCF within a degradable GelMA hydrogel matrix. Convective mixing of different precursor solutions of hydrogels was applied to creat a gradient environment in 3D hydrogels, which could influence cell aggregation and migration, or produce channels to transfer growth factors in gradient concentrations. By adjusting the geometry and the mixing models of the channels, they intend to use the device to develop a complex 3D culture environment that more closely mimics the elements of cell populations, matrix, and biological molecules within the BM. They observed single LSKs and Lin^+^ BM cells and found that using the spectral deconvolution methods could identify the LSKs (cKit^+^ Sca1^+^) from mature BM (Lin^+^) cells. The covalent incorporation ensured that the GelMA hydrogel retained its SCF content for longer than 7 days. The stable provision of this critical growth factor was sufficient to support the proliferation and lineage differentiation of HSCs.

In summary, modifying 3D hydrogels through the incorporation of important functional biomolecules *via* chemical or physical means can create a complex growth environment for HSC culture that more closely approximates the natural niche and allows the precise regulation of cell behavior to achieve extensive expansion of cells, while reducing undesired differentiation.

### 4.3 Regulating the biophysical features

Several groups have reported the beneficial effects of the biophysical characteristics of hydrogels during the culture of MSCs and embryonic stem cells (Lu et al., 2014; Wong et al., 2021; Costa et al., 2022). Based on prior experimental observation, hydrogels with tunable biophysical characteristics were developed to mimic the native spatial architecture of the hematopoietic niche (Zhang P. et al., 2019; Wong et al., 2021). The most important physical properties include the matrix architecture of the microenvironment, which can be appropriately modified by engineering changes (Vining and Mooney, 2017; Zhang Y. et al., 2019). The behavior of HSCs can be regulated by adjusting the stiffness, dimensionality, architectural features, viscoelasticity, and shear forces in hydrogel matrices.

Physiological compounds of the HSC niche vary in stiffnesses; for example, the stiffness of soft marrow or adipose tissue is lower than 1kPa, cell membranes is 1–3 kPa, and non-mineralized bone is >34 kPa (Patel et al., 2005; Discher et al., 2009; Chowdhury et al., 2010). Choi and Harley (2012) fabricated a polyacrylamide (PA) hydrogel substrate coated by collagen with varying stiffness to investigate the effect of substrate stiffness on the HSC behaviors. Elastic moduli of the PA hydrogel were adjusted by changing the ratio of the acrylamide and bis-acrylamide polymers to obtain a suitable cross-linking degree. They found that within the stiffness range of 0.01480–0.0442 kPa, varying the stiffness of the substrate, by altering its density, improved the viability of HSCs. The shape and spread area of 32D cells showed a similar trend to than of primary HSPCs when cultured on hydrogel with different stiffness. However, when the stiffness exceeded this range, the viability of the cells decreased with increasing substrate density. Additionally, the spreading of HSCs was enhanced significantly with the increased stiffness of the substrate. The effect of receptor-ligand interactions on HSPC behavior was also quantified, and it was observed that substrate stiffness significantly impacted the cytoskeletal distribution and density of HSPCs. Therefore, accurate control of hydrogel stiffness is important to ensure cell viability. Furthermore, HSCs cultured in a 3D hydrogel environment preserved their naïve state and rounded morphology better than when maintained on top of a 2D surface composed of the same components.

Hydrogel scaffolds containing macropores approximate the “spongy” architecture of natural trabecular bones. The resulting complex internal structure replicates the natural ECM of the hematopoietic niche and promotes the uniform diffusion of soluble factors by regulating their concentration and depletion (Raic et al., 2014). The pores can also aid the propagation of autocrine and paracrine signals involved in cell–cell communication (Raic et al., 2014; Gilchrist et al., 2019a; Gilchrist et al., 2019b). In one study, the poroelestic properties and Young’s modulus (E) of the GelMA hydrogels were controlled by adjusting the photo-initiator concentration and degree of methacrylamide functionalization (DOF). HSPCs and MSCs were both extracted from the tibia and femur BM of mice and were co-cultured in GelMA hydrogels with a high E value. After 7 days of culture, the hematopoietic population increased to the 11.11 ± 3.18% of the total, which is higher than that of a hydrogel with a low E value. This may be related to reduced myeloid differentiation of the cells. The percentage of quiescent (G0) LT-HSCs or HSCs to the initial HSPCs was highest for cells co-cultured in high E value hydrogel. This demonstrates that autocrine and paracrine signaling can be modulated by tuning the seeding density and poroelastic properties of the hydrogel matrix. Small mesh-sized pores resulted in slow diffusion, which reduced cell-to-cell communication, increasing the importance of autocrine regulatory dominated pathways. Under these conditions the quiescence of the primitive HSC population was better preserved (Gilchrist et al., 2019).

The viscoelasticity of hydrogels means that the stress relaxation characteristics of the matrix can be influenced by external pressure. These changes force cells to remodel the matrix through cell–ECM interactions (Tang et al., 2021). Chaudhuri et al. (2016) developed alginate hydrogels with tunable viscoelastic properties to adjust the differentiation of MSCs by modulating the nanoscale architecture of the matrix. They reported that at an elastic modulus of 17 kPa, the hydrogel showed fast stress relaxation and promoted MSCs to differentiate, resulting in the production of a mineralized collagen-1-rich matrix that showed some similarities to the structure of natural bone. Regulation of viscoelasticity can also affect mechanical stresses and substance diffusion (Chaudhuri, 2017). However, to date, most studies have relied on controlling the elasticity of the hydrogel during its preparation, rather than the adjusting the viscoelasticity dynamically (Chaudhuri et al., 2016). The modulation of hydrogel viscoelasticity may provide new insights to aid in the creation of an optimal microenvironment for the growth of HSCs. In the 3D hydrogel microenvironment, liquid movements result in a shear force being exerted over the HSCs (Rodell et al., 2013). Furthermore, hydrogel-based microfluidic systems are in development for high throughput cultures of HSCs. As microdroplets are formed in these systems, shear forces will change (Xia et al., 2017; Zhan et al., 2022). Studies have shown that shear stresses within the hydrogel suspension could reduce the survival of HSCs (Hosseinizand et al., 2016). Mahadik et al. (2014) fabricated a microfluidic multicellular gradient hydrogel platform and observed that the viability of MSCs and HSCs was not significantly influenced by flow characteristics. HSCs are exposed to a certain degree of shear force in their natural niche, so under ideal conditions this would need to be recreated in artificial matrices. Thus, shear forces in hydrogels need to be adjusted to a point where they support cell growth rather than reduce viability.

Zwitterionic materials have attracted attention recently due to their structural similarity to naturally occurring cell membrane lipids. Bai et al. (2019) developed zwitterionic polycarboxybetaine-based hydrogels modified with polypeptide cross-linkers. These exhibited enhanced hydration and swelled up markedly in aqueous solutions, which had an anti-fouling effect and improved biocompatibility (Figure 3C). HSCs cultured in this optimized zwitterionic hydrogel could undergo massive expansion without loss of their stemness (Figure 3D). A systematic study on expansion of primitive human HSCs using a zwitterionic hydrogel reported the construction of a uniform 3D zwitterionic hydrogel network (hereinafter referred to as ZTG hydrogel) through the click chemical reaction between alkyne terminated four arm polycarboxylated betaine acrylamide (pCBAA) and azide modified polypeptide polymerization (Azide–GG-(KE) 20-GPQGIWGQ-(KE) 20-GG Azide) to simulate the 3D environment dominated by hydrophilic and zwitterionic cell membrane lipids of HSPCs *in vivo*. ZTG hydrogel maintained the high expression of CD34 during the whole culture period, in which 93.7 ± 2% of the expanded cells were CD34^+^. Substantial expansion of primitive CD34^+^ BM- and CB-derived HSPCs for 24 days achieved 73-fold of increase in the number of LT-HSCs. The researchers also used limited dilution analysis (LDA) to detect the amplification frequency of medium and LT-HSCs; after 24–30 weeks of transplantation, the amplification frequency of LT-HSCs cultured in ZTOpt mode was much higher than that of other culture methods. Both LDA and secondary transplantation experiments showed that LT-HSC cells with long-term hematopoietic function could be cultured and expanded by ZTG. The LT-HSCs expanded in ZTG hydrogel were injected into immune-deficient NSG mice for primary and secondary BM transplantation. The experimental results showed that in HSPCs derived from CB, CD34^+^ was effectively amplified in ZTG hydrogel; the expanded HSPCs were used for BM transplantation, which also showed good amplification performance *in vivo*.

In the past, strategies for the *in vitro* expansion of HSCs mostly focused on the role of the ECM, while the effects of the metabolism of the HSCs themselves has received very little attention (Kohli and Passegue, 2014). Reactive oxygen species (ROS) produced by HSCs during proliferation is one of the key factors causing overactivation of the cells and results in the loss of hematopoietic potential (Suda et al., 2011). Incorporating ROS scavenging components into hydrogels represents a novel approach to maintain the quiescent status of HSCs. Gilchrist et al. (2021) encapsulated difficult-to-preserve HSPCs in a maleimide-functionalized gelatin (GelMAL) hydrogel containing added dithiol cross-linkers to absorb ROS. Bai et al. (2019) prepared 3D zwitterionic hydrogels that were unique in suppressing excess ROS production by inhibiting oxygen-related metabolism. Cells maintained in this environment showed reduced differentiation, thereby promoting their self-renewal.

### 4.4 Biomimicking cell–cell interactions

Cell–cell interactions within the hematopoietic niche provide both soluble factors and direct cell–cell contact, which are critical in determining the fate of HSCs (Walenda et al., 2010). Although supplementation with a cocktail of cytokines can increase the expansion of CD34^+^ HSCs (Li et al., 2019), cell–cell interactions during the co-culture of HSCs and MSCs can synergistically enhance cytokine-induced cell proliferation (Schmal et al., 2016). These observations prompted experiments in which various niche cells, such as MSCs and endothelial cells, were co-cultured with HSCs to investigate how the presence of another cell population affected the maintenance of stemness or the induction of desired lineage differentiation (Xiao et al., 2022). The results showed that MSCs and endothelial cells are the most important feeder cells in HSC cultures, as these cells produce more supporting factors than other BM stromal cells (Xiao et al., 2022). Although MSCs are relatively rare in the BM, they secrete several critical cytokines and growth factors, including CXCL12, which direct chemotaxis, survival, proliferation, and promote SFC. Furthermore, key differentiation factors, such as Flt3-ligand, TPO, and IL-6, are all produced primarily by BM-MSCs (Lo Iacono et al., 2017; Sarvar et al., 2022). Human umbilical vein endothelial cells (HUVECs) have also been used as feeder cells to support the proliferation and differentiation of HSCs *in vitro*. They express some of the same surface markers and transcription factors found in HSCs, including CD31, CD34, Runx1, and GATA-2 (Kuan et al., 2017; Li et al., 2019).

Co-culturing HSCs with MSCs or HUVECs provides important cell–cell interactions. However, because both MSCs and HUVECs are adherent cells, their interactions with HSCs on a 2D surface are suboptimal (Bello et al., 2018). To improve the cell–cell interactions in co-culturing, hydrogels were used to create an artificial matrix in which adhesion molecules present on MSCs and HUVECs interacted in a 3D spatial area with the HSCs, resulting in improved HSC proliferation (Tibbitt and Anseth, 2009).

Leisten et al. (2012) reported a collagen-based 3D microenvironment for co-culture of human BM or umbilical cord derived progenitor cells (HPCs) with MSCs. MSCs were embedded in the collagen hydrogel (denoted as niche II) and HPCs were seeded in the collagen-free suspension (denoted as niche I). After 14 days culture, HSCs migrated along the collagen fibers niche II and formed cobblestone-like cluster of 10 cells. After 14 days of culture, HPCs in the BM-MSC contained in niche II had a highest number of CD34^+^ cells, while the lowest number of CD34^+^ cells was observed in umbilical cord (UC)-MSC niche II, indicating that UC-MSC stimulated HPC differentiation. In niche II, expression of the pan-leukocyte marker CD45, and CD13 were observed, whilst CD56 was poorly expressed in niche II and myeloid differentiation was increased. The researchers established a collagen hydrogel-based artificial HSC niche with two statuses: HPCs in niche I are a proliferative and contained cell population of CD34^+^/CD38^-^ maturing myeloid cells (CD38^+^, CD13^+^, CAE^+^), while HPCs in niche II contained high numbers of primitive CD34^+^/CD38^-^ phenotype cells beginning myeloid (CD13^+^, CAE^+^) differentiation, resembling the endosteal part of the BM niche. These authors also reported that UC-MSCs were not appropriate feeder cells as they caused extensive differentiation and a complete loss of primitive HSC phenotype (Leisten et al., 2012). Hydrogels can contain a complex diffusion system with an anisotropic distribution of molecules that is more similar to the natural niche, potentially allowing a more precise regulation of cellular behavior (Gurkan et al., 2014). Tuning of the mesh size in hydrogels can precisely regulate cell–cell interactions between co-cultured HSCs and MSCs; this approach allows the balancing of autocrine and paracrine signals (Gilchrist et al., 2019). The co-culture of Lin^+^ BM niche cells and HSCs in different hydrogel matrices showed that proliferation rate and myeloid differentiation of HSCs closely depended on the rate of diffusion in the matrix (Mahadik et al., 2017). Integrating hydrogels with microfluidic technology resulted in a novel 3D artificial hematopoietic niche (Carreras et al., 2018; Carreras et al., 2021). In this double-layered model, alginate embedded MSCs were place in the middle of hydrogel beads. This core was surrounded by an outer layer of Puramatrix encapsulated HSCs. Figures 3E,F HSCs embedded in these double layered beads maintained their stemness even after 8 weeks of culture (Figure 3G).

### 4.5 Optimization: Integrating functional nanomaterials

Organic polymers used as hydrogel sources have distinct advantages due to the superior biocompatibility of their natural matrix (Guvendiren and Burdick, 2013). Unfortunately, these polymers lack the necessary physical features and properties to create a viable culture system (Billiet et al., 2012). Recently, inorganic nanomaterials with excellent mechanical strength, abundant functional groups, good optical properties, and a large surface area have gained increasing use in tissue engineering (Billiet et al., 2012). To improve the mechanical properties and extend the application of biologically derived hydrogels in the production of cultured HSCs, organic–inorganic 3D hybrid hydrogels were prepared *via* the physical or chemical cross-linking of biopolymers with functional nanomaterials in an aqueous phase (Motealleh and Kehr, 2017). These nanomaterial-containing hybrid hydrogels exhibited improved mechanical, electronic, and optical characteristics. Their stimulus-response properties and biocompatibility are also very promising (Gaharwar et al., 2014; Kehr et al., 2015; Motealleh and Kehr, 2017; Sharma et al., 2018; Guillet et al., 2019; Deng et al., 2021).

Zhang P. et al. (2019) fabricated a HAMA hydrogel *via* the esterification of MA and HA. The macromolecular ROS scavenger, carbon nanotubes (CNTs) were loaded into the HAMA hydrogel by mixing the CNTs suspension with HAMA and LAP in pre-gel solution and followed by a photo-cross-linking. CNT hybrid hydrogels showed better hydrophilicity because of the abundant carboxyl groups from the CNTs. CNTs also improved the modulus of the hydrogels from 5.0 to 7.5 kPa. Isolated CD34^+^ cells were cultured on the hydrogels supplied with the cytokine cocktail. HSC release of ROS decreases the survival of HSCs and causes the activation of HSCs. In the CNT-loaded HAMA hydrogel, ROS triggers the biodegradation of polysaccharides in the hydrogel, releasing the CNTs to scavenge ROS. The percentage of CD34^+^CD38^−^ and CD34^+^ cells increased in the CNT hybrid hydrogel after 10 days of culture. CNT hybrid hydrogels also significantly promoted the total CFU, CFU-GM, and CFU-GEMM compared with non-CNT loaded hydrogels, indicating that integrating nanomaterials with antioxidant properties is beneficial for the hematopoietic function of HSCs. Another study combined methacrylated graphene oxide (MeGO) with HA hydrogels, which significantly increased the water absorption and elastic modulus of the hybrid material. The composite MeGO-HA matrix supported the proliferation of CD34^+^ cells, whilst maintaining their primitive CD34^+^CD38^−^ phenotype, especially in a version containing 0.05% w/v MeGO (Guo et al., 2017). To promote the differentiation of HSCs into red blood cells, Brandle et al. (2020) developed an iron nanoparticle composite PEG hydrogel, which ensured the gradual release of iron, inducing some erythroid differentiation. However, they failed to achieve the desired level of differentiation due to iron overload in the 3D PEG-iron nanocomposite scaffolds.

## 5 Fabrication technologies to create a hydrogel-based artificial niche

### 5.1 Hydrogel coating on 2D substrates

Culturing HSCs on the 2D surface of polystyrene flasks is still the most widely used traditional culture method (Ribeiro-Filho et al., 2019). This approach has low costs, the flasks are easy to handle and the cells can be harvested with ease (Bello et al., 2018). However, the hydrophobicity of these plastic substrates can induce attachment and the excessive production of ROS, resulting in unintended differentiation and the rapid loss of stemness (Bai et al., 2019; Erathodiyil et al., 2020). It has been demonstrated that culturing cells on plastic surfaces coated with hydrophilic hydrogels inhibited this unnecessary differentiation (Cuchiara et al., 2016; Bai et al., 2019; Liu et al., 2021). Choi and Harley prepared type I collagen-coated polyacrylamide substrates of varying stiffness by altering the total polymer content and the density of cross-linkers. The spreading and morphology of HSCs was significantly influenced by the 2D hydrogel coating of the polyacrylamide surface (Choi and Harley, 2012). However, using a 2D environment for the culture of HSCs is substantially different from the 3D architecture of their natural niche. This leads to a non-physiological flattened spreading pattern with abnormal polarity that causes the loss of their multilineage differentiation potential (Caliari and Burdick, 2016). Compelling evidence suggests that 3D systems enhance cell-microenvironment interactions by improving mechano-sensing by HSCs resulting in increased proliferation and the maintenance of repopulating capacity even after long periods in culture (Cuchiara et al., 2016; Gvaramia et al., 2017; Gilchrist et al., 2019). Thus, current research trends are focusing on the development of optimized 3D hydrogel systems (Jensen and Teng, 2020).

### 5.2 3D hydrogel encapsulation

To better mimic the 3D hematopoietic microenvironment, the encapsulation of HSCs into a synthetic 3D hydrogel space has been extensively studied (Bello et al., 2018). As mentioned above, these hydrogels can contain natural or synthetic polymers, or a combination of the two. Some of the most commonly utilized natural hydrogels include HA, collagen, alginate, and chitosan, while synthetic hydrogels commonly include PEG, polyethylene oxide (PEO), and poly-l-lactic acid (Maji and Lee, 2022).

Traditional bulk hydrogels are usually relatively large and lack micropores, resulting in slow degradation and limited substance diffusion (Hsu et al., 2019). Micron-sized hydrogels, termed microgels, are expected to solve these problems. These microgels can act as a culture unit for a single cell or may contain a co-culture of multiple cells. Alternatively, the hydrogel can also be assembled into larger scaffolds (Carreras et al., 2018; Caldwell et al., 2020; Carreras et al., 2021). Carreras et al. (2021) reported the use of a droplet microfluidic device contained with double-layered hydrogel beads for the long-term culturing of human HSCs (CD34^+^ cells). They presented a 3D niche biomimetic model by fabricating an engineered double-layered bead with an alginate-based inner layer and a Puramatrix-based out layer. Numbers of CD34^+^ cells significantly increased at weeks 6–7, reaching to a maximum level at week 7 of approximately 4-fold that of week 1–2; it then gradually declining at week 8. At week 4, most cells were functional and remained in an undifferentiated status; however, there was a slight CD20 and CD38 signal for lymphoid differentiation, and CD33 signal for myeloid differentiation, as well as CD34^+^/HLA^−^DR^+^ for an intermediate stem cell population. The results indicated this biomimetic niche model had good potential for the long-term culture of HSCs and maintenance on functional cell numbers.

The above methods enabled the microencapsulation of co-cultured HSCs and MSCs with a population distribution approximating that of the natural niche. Encapsulating cells in 3D hydrogels has been demonstrated to regulate stem cell fate decisions by supporting tunable ligand incorporation, the delivery of soluble factors, and mechano-sensing (Gvaramia et al., 2017; Kim et al., 2019; Gilchrist et al., 2021).

### 5.3 Hydrogel-integrated microfluidic systems

Microfluidic technology has emerged as a powerful tool in the reconstruction of the cellular microenvironment. This approach enables the creation of micro-sized droplets matching the diameter of the cells and permits the precise microscale manipulation of fluid flow and the modulation of the concentration of various factors (Whitesides, 2006; Sackmann et al., 2014; An et al., 2019). However, conventional microfluidics manipulations usually take place in 2D environments (An et al., 2019). Incorporating hydrogel matrices into microfluidic systems enables the construction of 3D spatial environments and the provision of different biophysical and biochemical features mimicking the natural microenvironment (Liu et al., 2019).

The use of microfluidic systems can rapidly establish a concentration gradient of various chemical compounds, facilitating the precise control of culture conditions and experimental investigation of the effects of individual factors (An et al., 2019). Chemical gradients are created in microfluidic channels *via* both flow and diffusion (Kim et al., 2010). T- or Y-shaped channels also allow the mixing of two fluid streams entering from two separate inlets and converging at the intersection (An et al., 2019). Mahadik et al. (2015) reported the use of the PEG-SCF functionalized GelMA hydrogel; PEG functionalized SCF retains the natural biological activity of SCF, and >80% of PEG functionalized SCF can be stably incorporated and retained in the GelMA hydrogel within 7 days. Mouse BM HSCs were cultured in hydrogels with four SCF presentation modes (No SCF, transient SCF, continuous SCF, concurrent SCF). The effects of continuous and covalent SCF presentation patterns on the fate of HSCs were examined by surface antigen expression and CFU analysis. After 7 days, the total number of cells under both conditions increased significantly; however, the maintenance of LSK and CD34^−^LSK components decreased significantly under continuous SCF conditions, while in the hydrogel containing covalently fixed SCF, they remained unchanged over time. In GelMA hydrogel containing covalent fixation, the CFU-GEMM score was significantly higher than that of continuous SCF, indicating that the maintenance of more primitive hematopoietic progenitor cell population was improved. They also mixed two pre-polymer solutions (5% w/v GelMA; 5% w/v GelMA ^+^ 400 ng/ml PEG-SCF) through a microfluidic device using a computer-controlled syringe pump to form a GelMA hydrogel containing a covalently fixed SCF gradient, and observed that HSCs expanded with the SCF content gradient. SCF embedded in this 3D environment promoted the proliferation and the maintenance of primitive HSCs in a dose-dependent manner.

In addition to chemical gradients, microfluidics can also generate cellular gradients to mimic the complex multicellular microenvironment. Mahadik et al. (2014) used a traditional staggered herringbone shaped microfluidic mixer to generate a 3D collagen hydrogel containing adjustable, reverse gradient cells and biomaterial properties from two hydrogel precursor suspensions. To check the effect of flow through the mixer on cell viability, a 1-mg/ml collagen suspension containing MC3T3-E1 osteoblasts or mouse BM LSK cells was directly put into the glass dish (control) or entered the dish through the microfluidic mixer (device). For these 2 cell types, high relative viability was observed in the first 24 h of culture, which remained high after 3 days of culture (>60%). The mixer was then used to prepare the reverse gradient hydrogel of the above 2 cells in the 1 mg/ml collagen hydrogel. The relative gradient (2:1–1:2) of LSK and osteoblasts was quantified by fluorescence analysis of the whole structure. The relative gradient of mixed cells was similar to that of single cell hydrogel with opposite gradient, indicating that the relative gradient of mixed cells was generated. Single LSK and Lin^+^(GFP^+^) BM cells mixed in a single hydrogel were imaged using a multiphoton microscope. The spectral deconvolution method could identify LSK cells from mature BM (Lin^+^) cells. The multiple gradient hydrogel constructs developed here provide the potential for HSC fate manipulation and tracking analysis in 3D microenvironment models, and will become a powerful tool for studying cell–microenvironment interactions (Choi et al., 2015).

The human organ chip is a transformative biomedical technology that was voted as one of the “Top Ten Emerging Technologies” in 2016 (Domingues et al., 2017). In one application, scientists established a BM on-a-chip based on the integrations of hydrogels and a microfluidic system. The hydrogel element contained various encapsulated niche cells (Sieber et al., 2018; Chatterjee et al., 2021). This BM on-a-chip approach represents a sophisticated model of the living BM with cellular diversity and complex functionality, and was subsequently approved by the Food and Drug Administration (Liu et al., 2019). BM toxicity, radiation resistance, and drug testing can be conducted using these artificial BM models, replacing experimentation on animals (Chou et al., 2020).

**FIGURE 4:**
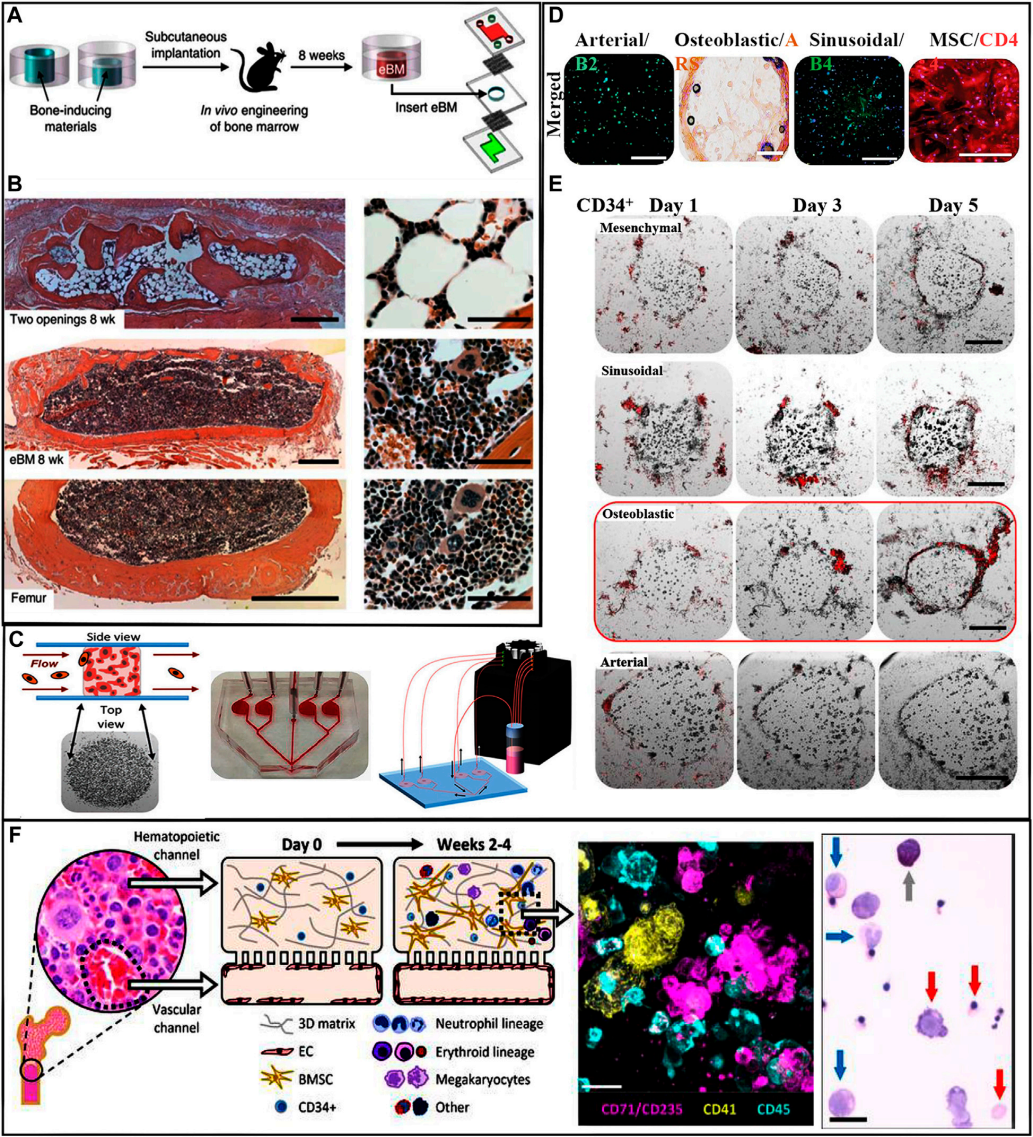
**(A)** Schematic of workflow to fabricate BM on-a-chip, in which eBM is formed in mouse and is then cultured in a microfluidic system. **(B)** Images show the establishment of eBM with in vivo-like histological BM structure. (Top) Hematoxylin-and-eosin-stained sections of the post-implanted eBM formed in the PDMS device with two opening. (Center) Histological sections of the post-implanted eBM formed in the PDMS device with one lower opening. (Bottom) Cross-section of BM in a normal adult mouse femur. Scale bars: 500 and 50 µm for low and high magnification views, respectively. (Torisawa et al., 2014) Figure 4A, B: Copyright © 2014. Reproduced with permission from Springer Nature. **(C)** Niche-on-chip microfluidic chip. (Left) Side and top view depictions of the niche constructs in the device chambers. (Center) Schematic of multi-niche-on-chip device. (Right) Schematic of operational recirculating multi-niche-on-a-chip systems. **(D)** Live/dead viability staining of each niche construct type. Scale bars: 100 µm. **(E)** Fluorescent images of niche-on-a-chip lodging/retention experiments using CD34+ HSCs. Scale bar: 250 µm. (Aleman et al., 2019) Figure 4C-E: Copyright © 2019. Reproduced with permission from John Wiley and Sons. **(F)** Schematic illustration of the vascularized BM on-a-chip with CD34+ progenitors and MSCs in the top gel channel and a vascular lining in the bottom channel. Immunofluorescence image of a vertical cross-section through the upper channel of the BM chip. Scale bar: 20 µm. Wright-Giemsa staining image of cells from the BM chip showing multilineage differentiation. Scale bar: 20 µm. (Chou et al., 2020). Copyright © 2020. Reproduced with permission from Springer Nature.

These organ on-a-chip models closely approximate the environment of the natural BM. Torisawa et al. (2014) developed a BM on-a-chip system and tested whether HSCs cultured on these chips can maintain their capacity to self-renew and differentiate into multiple lineages. They fabricated a polydimethylsiloxane microdevice contained a central cavity filled with collagen, bone morphogenetic proteins, and demineralized bone powder. These devices were implanted into mice for a period of 4–8 weeks. On removal, the central cavity contained a well-developed BM compartment. These recovered BM chips were transferred into a microfluidic system for further observation (Figure 4A). After 7 days of *in vitro* culture, the amount and distribution of HSPCs and HSCs remained similar to those of the fresh BM (Torisawa et al., 2014) (Figure 4B). Aleman et al. (2019) created another version of the BM on-a-chip; the HA and gelatin hydrogel microenvironment was subdivided into four major hematopoietic areas, a periarterial, a perisinusoidal, a mesenchymal, and an osteoblastic niche (Figures 4C,D). In this system, healthy and malignant human HSCs showed unique and selective homing behavior into specific niches (Figure 4E). Chou et al. (2020) created a vascularized human BM on-a-chip system that consisted of two chambers connected by a porous membrane. The top “hematopoietic” chamber, representing the intraosseous niche, was filled with human CD34^+^ HSCs and BM-derived MSCs in fibrin gels. The bottom “vascular” chamber was lined by HUVECs and perfused with culture medium containing supportive cytokines (Figure 4F). Culturing HSCs within this *in vitro* model preserved stemness and supported myeloerythroid proliferation and differentiation (Chou et al., 2020). Research on the validation of the effectiveness of HSCs developed in these artificial environments is currently being carried out in living animals. The HSC populations expanded in the engineered environments *in vitro* are transplanted into mice for evaluation of their safety and their ability to reconstitute BM function (Baena et al., 2021). It is anticipated that future BM on-a-chip systems will be sophisticated enough to allow the validation and characterization of *in vitro* expanded HSCs without the need to sacrifice live animals.

### 5.4 Stimulus-responsive hydrogel-based multifunctional hematopoietic stem cell culture platform

Stimulus-responsive hydrogels are promising tools that can sense signals coming from niche cells and/or cytokines in the matrix and respond to these signals by changes in conductive, chemical, or temperature characteristics. These “intelligent” microenvironments can adapt to the requirements of the cells growing within (Jiang et al., 2020; Deng et al., 2021). For example, an enzyme-responsive 3D hydrogel was developed containing conjugated polypeptides that could be hydrolyzed by the matric metalloproteases (MMPs) secreted by encapsulated HSCs. As HSC numbers expanded, their increased cumulative MMP production gradually cleaved the polypeptides in the matrix, facilitating the swelling of the hydrogel, thus providing a larger space for further increases in HSC number. In addition, the enzyme sensitive nature of the hydrogel also simplified the rapid harvesting of cells. By exposing the whole hydrogel compartment to exogenous MMPs, this resulted in the degradation of the matrix, so the encased cells could be easily collected (Bai et al., 2019). Although hydrogel-based 3D environments allow the successful expansion of HSCs, harvesting the encapsulated cells remains challenging (Bodenberger et al., 2017; Yin and Cao, 2021). Commonly the expanded cells are collected after mechanical disassembly or chemical dissociation of the matrix. However, these processes can damage the cells. Kwak and Lee, 2020 reported an inverted colloidal crystal hydrogel scaffold that was created from poly(N-isopropylacrylamide). The volume of this hydrogel shrank or swelled significantly depending on changes in the temperature within a physiological range (4–37°C). Due to this reversible thermoresponsive behavior of the hydrogel spheroids containing embedded cells could be created in a single step and the expanded cells could be rapidly recovered afterwards by controlling the volume of the hydrogel. By changing the temperature, the encapsulated HSCs could be selectively separated from spheroids.

Stimulus responsive hydrogels can be combined with biosensor technology to directly monitor key markers of living cells cultured in the biomimetic environment. This approach allows for the long-term tracking and monitoring of the encapsulated cells (Jiang et al., 2020; Deng et al., 2021). Lee et al., 2016 reported a 3D capacitance biosensor that worked in alginate hydrogels containing MSCs. This system allowed the real time monitoring of cell proliferation, migration, and differentiation, by measuring dynamic changes in capacitance and conductance. Because HSCs can easily lose their stemness during in vitro culture, the ability to monitor their differentiation and stemness markers, preferably in real time, has considerable advantages (De Leon et al., 2020; Allenby and Woodruff, 2022).

While current hydrogel methods enable the massive expansion of encapsulated cells, they are seldom equipped to sense and monitor cell characteristics (Fedi et al., 2022). Instead, the detection of biomarkers still relies on conventional fluorescence or immunological detection techniques requiring the extraction of cells from the matrix. Under these conditions, the real-time state of the bionic culture environment cannot be continuously monitored (Lim et al., 2007; Gilchrist et al., 2021). Stimulus-responsive hydrogels will provide new approaches in developing a comprehensive platform for the expansion and simultaneous monitoring of the state of encapsulated HSCs, potentially optimizing yield and quality.

## 6 Prospects and challenges

Hydrogel biomaterials show excellent biosafety, offer flexible possibilities for chemical modifications and biofunctionalization, and have been successfully used for the *in vitro* expansion of primitive HSCs. A combination of various chemical modifications, the addition of biomaterials, and advancements in technologies—such as microfluidics and organ on-a-chip—make it possible to establish hydrogel-based microenvironments with exquisite architecture and adjustable physical features that closely mimic the natural hematopoietic niche. Aside from their clinical relevance, these systems also facilitate studies on the behavior and fate of HSCs. However, due to the extraordinarily complex physiology of the natural hematopoietic niche, replicating it *in vitro* using engineered hydrogel-based systems still holds many challenges. Various features of the natural hematopoietic environment, such as the presence of other cells, biochemical cues, varying stiffness, and topography, are distributed in a heterogeneous environment and direct the behavior and fate of HSCs though multiple mechanisms. In contrast, most hydrogel-based culture systems exhibit isotropy and lack the anisotropic properties of the tissue microenvironment. Although the integration of hydrogels in microfluidic systems enabled the dynamic co-existence of two distinct cell populations, the gap between these and the natural niche remains wide. BM on-a-chip technology is a promising and powerful tool for *ex-vivo* HSC culture and the monitoring and scientific exploration of the hematopoietic process. However, in their current iteration, they can only accommodate no more than two distinct cell populations. In addition, their complex manufacturing and high costs currently limit their large-scale production for clinical applications.

In general, hydrogel have uniform internal structures, which differs significantly from the architecture of a natural niche (Zimmermann et al., 2019). 3D printing of biomaterials is powerful emerging tool for the fabrication of sophisticated and highly organized 3D hydrogel structures (Ong et al., 2018; Olate-Moya et al., 2020; Moore et al., 2021). Composite hydrogels with enhanced shape fidelity can facilitate the printing of scaffolds with mechanical and architectural details closely resembling the features of the *in vivo* niche (Zhou et al., 2020). A composite hydrogel composed of 4% methylcellulose and 2% alginate that also contained encapsulated BM niche cells was used to bioprint a scaffold to mimic the perivascular area of the BM (Moore et al., 2021). However, 3D printing for artificial niche mimicking is a new technology that requires further exploration.

The natural hematopoietic niche contains a viscoelastic matrix, while most hydrogels are primarily elastic materials. These artificial matrices cannot relax under strain forces. In contrast, the natural viscoelastic matrix is mechanically yielding, and the matrix can be dynamically remodeled. The development of better viscoelastic hydrogels, replicating the conditions for cell–ECM interactions *in vivo*, could significantly improve the outcomes of culturing HSCs *in vitro*.

## 7 Conclusion

HSCs from CB play an important role in clinical transplantation. CB provides an alternative source for allogeneic HSCT patients lacking HLA-matched donors (Rocha, 2016). Compared with BM and mobilized peripheral blood stem cells, CB has lower requirements on HLA matching, increased donor availability, and low rates of chronic GVHD. However, due to the limited number of HSCs and HSPCs within a single umbilical CB unit, the implantation of neutrophils is delayed, which increases the risk of early infection and transplant-related mortality. Therefore, increasing research has been devoted to the exploration of new techniques for functional HSC expansion *in vitro*. However, there are very limited to normal HSCs that undergo asymmetric mitosis. HSCs are very sensitive to the environment, which means after leaving the niche, stimulation from the external environment, such as mechanical force or redox reaction, can easily cause their differentiation and loss of the expression of original phenotype markers.

In the previous, Section 2, we discussed the complexity of the BM niche and the correlation of several elements of this niche in HSC physiology. Recent HSC culture methods such as adding cytokines, small molecule compounds and perfusion reactor in the laboratory, have been introduced. In addition, building a 3D culture environment that is closer to the HSC niche in terms of space and biological interaction has gradually become a new trend in the study of HSC expansion *in vitro*. The chemical and engineering strategies and techniques for improving the niche of biomimetic hematopoiesis were summarized. The potential of stimulus-responsive hydrogels in the establishment of an intelligent microenvironment was also described. Finally, the remaining challenges in the use of the hydrogel culture platform and the broad prospects for development were reviewed. The improved artificial microenvironment for HSC amplification *in vitro* and the bionic BM for testing the safety and effectiveness of HSC treatment are exciting developments, providing new solutions for the increasing use of hydrogel-based HSC culture platforms in clinical applications and scientific research. However, due to the complexity of the niche, in the current technology, it remains difficult to artificially reconstruct all these elements into functional BM. The development of an effective bioengineering strategy requires further research on the molecular mechanism of HSC behavior in the niche and the progression of biomaterial technology.
